# Effect of Role Ambiguity and Fatigue on Employee Performance in Pelamonia Hospital, Makassar, Indonesia

**Published:** 2020-01

**Authors:** Arlin ADAM, Andi ALIM, Dedy MAULANA

**Affiliations:** 1 Department of Health Education and Behavioral Science, Faculty of Public Health, Pejuang Republic of Indonesia University, Makassar, Indonesia; 2 Department of Health Policy Administration, Faculty of Public Health, Pejuang Republic of Indonesia University, Makassar, Indonesia; 3 Department of Health Promotion, Faculty of Public Health, Pejuang Republic of Indonesia University, Makassar, Indonesia

## Dear Editor-in-Chief

The existence of hospitals as public service organizations in the health sector is required to meet quality standards to provide satisfaction for the community. The aspect that determines hospital quality standards is employee performance. The importance of employee performance is that employee performance absolutely must be improved by the demands and needs of the Indonesian people (1). Factors that need to be considered by an organization are role ambiguity that has a negative and not significant effect on employee performance (2). Another factor is work fatigue that work fatigue weakens function and performance (3).

The variables affecting hospital performance in determinants were role ambiguity and work fatigue. These variables are then tested by assessing the performance of Pelamonia Makassar Hospital as the largest referral hospital for the military community in Eastern Indonesia. The research method was quantitative with statistical tests (*t*-test and F test) to measure the significance of the relationship between variables. The total population of 370 people with a sample of 76 people drawn by purposive sampling (Table 1).

**Table 1:** Distribution of respondents’ answers to the research variables

***Research Variable***	***Frequency***	***Percentage***
Role Ambiguity		
Low < 60%	71	89.9
High ≥ 60%	8	10.10
Work Fatigue		
Low < 60%	68	86.10
High ≥ 60%	11	13.90
Employee Performance		
Low < 60%	11	13.90
High ≥ 60%	68	86.10

Source: Primary data, 2019

Table 1 explained that employee experiencing low ambiguity more than high role ambiguity, while the level of work fatigue was found low fatigue level more than high fatigue level. Specifically for the performance variable, high performance was more than low performance. Respondents’ answers above have high levels of validity and reliability because the instrument used has been tested for validity with a correlation coefficient value exceeding 0.30 and the reliability test results in a Cronbach’s alpha value above 0.60 (4).

Multiple linear regression analysis found that without role ambiguity and work fatigue, employee performance amounted to 5.868, while the role ambiguity value was obtained by −0.334, meaning the higher role ambiguity affected employee work fatigue. The value of work fatigue variable obtained by the number −0.520 means that work fatigue affects the decline in employee performance. A statistical picture of these results is seen in Table 2.

**Table 2:** Processed Data Results in Multiple Linear Regression Coefficients between variables

***Model***		***Unstandardized Coefficients***		***Standardized Coefficients***	**t**	***Sig.***
		***B***	***Std. Error***	***Beta***		
1	(Constant)	5.868	.265		22.136	.000
	Role Ambiguity	−.347	.111	−.285	−3.141	.002
	Work Fatigue	−.520	.092	−.511	−5.639	.000

Source: Data processed, 2019

The multiple correlation coefficient (R) of 0.638 shows the relationship between role ambiguity and work fatigue with a performance of 63.8%. This means that if there is an unclear role of the employee and the work fatigue that is felt by the employee, it will affect the performance decline. The coefficient of determination R2 of 0.407 indicates a 40.7% relationship between variables while the remaining 59.3% represents the opportunity for involvement of other variables not examined as stated that four to five dimensions of patients, internal processes, financial dimensions, employees’ learning and growth, and clinical dimensions have been determined in the BSC model and can be useful in evaluating hospitals (5).

Table 3 regression testing shows the significant relationship between variables.

**Table 3:** Model summary of the relationship between variables

***Model***	***R***	***R Square***	***Adjusted R Square***	***Std. Error of the Estimate***
1	.638^a^	.407	.392	.74235
a.	Predictors: (Constant), Role Ambiguity, Work Fatigue			

Source: SPSS Appendix

There was a negative and significant effect between role ambiguity and performance with the *t*-value of −3.141> *t*-table = 1.665. While work fatigue and performance obtained the *t-*count value of −5.639> *t*-table = 1.665 showed there was a negative and significant effect means that if work fatigue increases, it will affect the performance. Meanwhile, the simultaneous test (f test) with a confidence level of 95% obtained a significant value of 0.000 <0.05 means that if the role ambiguity and work fatigue increase, it will affect the decrease in employee performance.

The conclusion of this study is that the role ambiguity has a negative and significant effect on performance and work fatigue has a negative and significant effect on the performance of employees at Pelamonia Hospital in Makassar.
